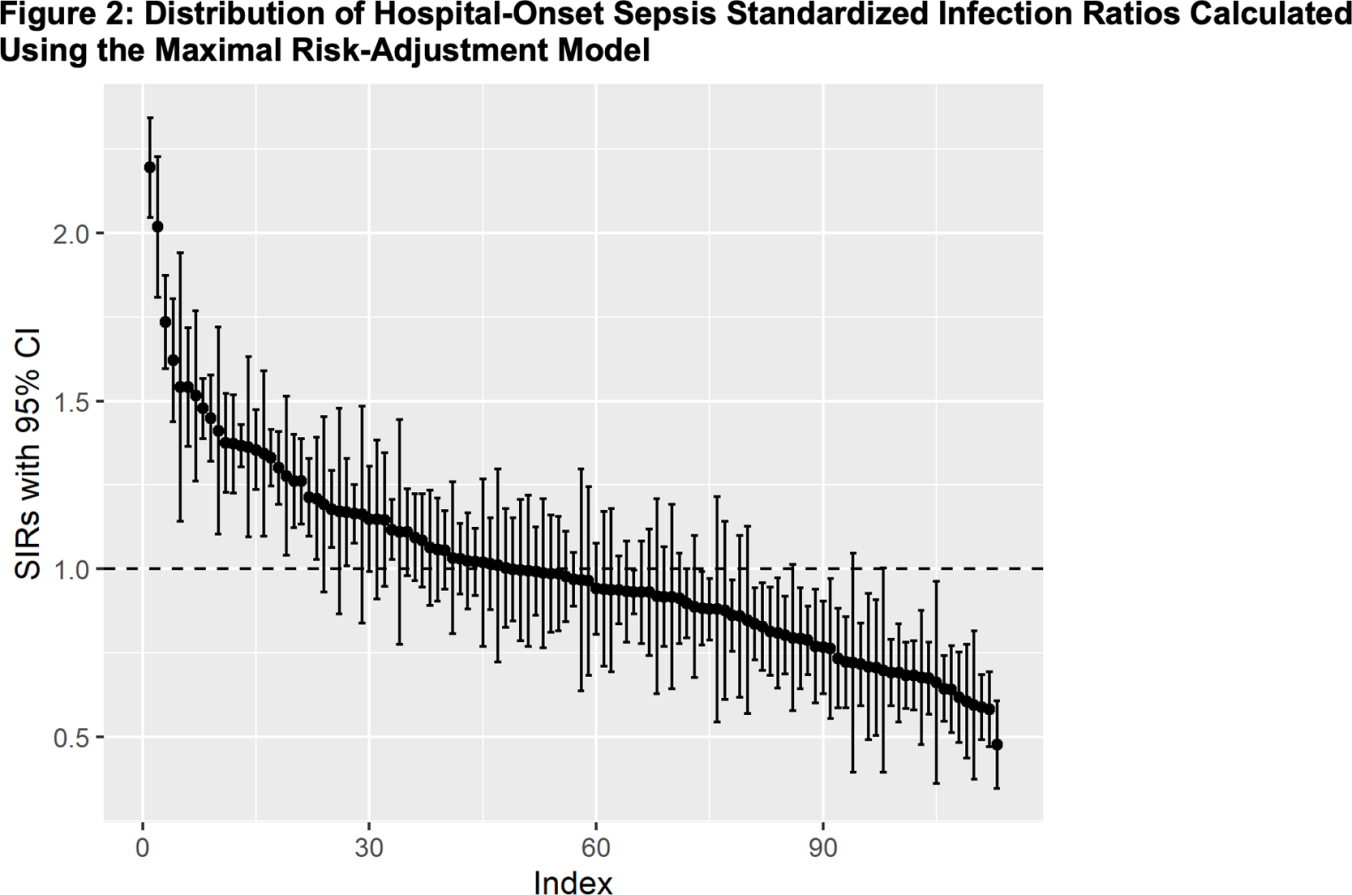# 126 Artificial Intelligence Photo-Detection of High-Risk Lines to Prevent Central Lines Associated Bloodstream Infection (CLABSI)

**DOI:** 10.1017/ash.2026.10458

**Published:** 2026-06-23

**Authors:** Fizza Manzoor, Tingting Yu, Michael Klompas, Robert Jin, Ed Septimus, Jefrey Guy, Ken Sands, Russell Poland, Julia Moody, Samir Fakhry, Matti Hautala, Micaela Coady, Candace Fuller, Kimberly Smith-Sells, Raymund Dantes, Rui Wang, Chanu Rhee

**Affiliations:** 1 Brigham and Women’s Hospital; 2 Harvard Medical School and Harvard Pilgrim Health Care Institute; 3 Harvard Medical School; 4 Frist College of Medicine, Belmont University; 5 HCA Healthcare; 6 Harvard Pilgrim Health Care Institute; 7 CDC / Emory University; 8 Brigham and Women’s Hospital / Harvard Medical School

## Abstract

**Background:** Electronic surveillance for hospital-onset sepsis using CDC’s Adult Sepsis Event definition could provide an efficient and objective method to identify a broad array of serious healthcare-associated infections, many of which are missed through current reporting processes. We developed risk adjustment models of varying complexity to support facility-level comparison of hospital-onset sepsis rates, evaluated trade-offs between model performance and feasibility, and quantified residual inter-facility variation that may reflect gaps in care. **Methods:** We conducted a retrospective study of adults hospitalized for <3 days within 113 community hospitals between 2022-2023. Hospital-onset Adult Sepsis Events (HO-ASEs) occurring on day 4 or later were identified using updated CDC surveillance criteria. We used logistic regression to develop three risk adjustment models of increasing complexity using covariates from administrative and electronic health record data: basic model (hospital and aggregate patient descriptors), intermediate model (replacing aggregate patient descriptors with patient-level descriptors), and maximal model (adding detailed physiologic and clinical data from hospital days 1-3; Figure 1). We evaluated model performance using Area Under Receiver Operating Curve (AUROC), calculated hospital-level Standardized Infection Ratios (SIRs) for each model and assessed concordance in hospital rankings using Kendall’s tau coefficient (τ). **Results:** The cohort included 1,557,252 hospitalizations of <3 days, of which 24,169 (1.6%) met HO-ASE criteria and 8,500 (35.2%) died in-hospital. The basic model had limited discrimination (AUROC 0.589, 95% CI, 0.585-0.593). Adding patient-level characteristics to form the intermediate model markedly improved performance (AUROC 0.839, 95% CI 0.836-0.841), with further inclusion of detailed clinical data in the maximal model yielding modest additional improvement (AUROC 0.850, 95% CI 0.848-0.853). Concordance between hospital rankings derived from the crude or basic risk-adjusted HO-ASE rates versus rankings derived from the intermediate or maximal models was moderate (τ 0.40-0.51) whereas concordance between rankings derived from the intermediate vs. maximal models was high (τ 0.86). There was a wide distribution of HO-ASE SIRs across facilities even after risk adjustment using the maximal model (Figure 2), with high signal-to-noise ratios and good calibration. **Conclusions:** Risk adjustment models incorporating hospital characteristics and patient-level data perform well and might explain substantial variability in HO-ASE rates between facilities. The persistence of residual variability after highly detailed adjustment may reflect differences in care processes, suggesting that risk-adjusted HO-ASE comparisons can help identify gaps and opportunities in the prevention of severe healthcare-associated infections. Our findings support the use of HO-ASE as an electronic, scalable, risk-adjusted metric for facility-level benchmarking to inform quality improvement initiatives.

**Figure:**
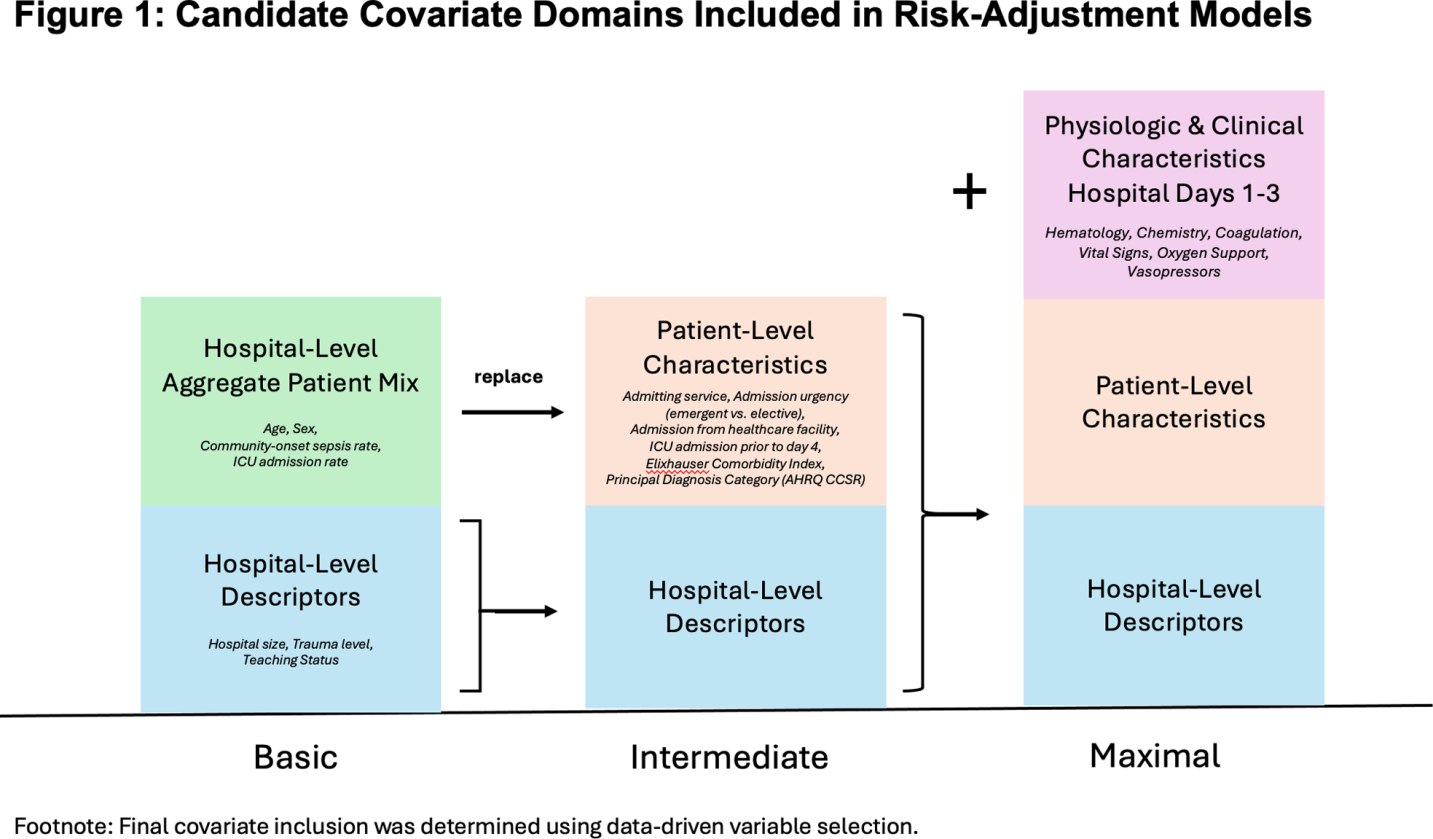

**Figure:**